# The Potential of Superoxide Dismutase-Rich *Tetraselmis chuii* as a Promoter of Cellular Health

**DOI:** 10.3390/ijms26041693

**Published:** 2025-02-16

**Authors:** Stuart P. Cocksedge, Lalia Mantecón, Enrique Castaño, Carlos Infante, Stephen J. Bailey

**Affiliations:** 1 School of Sport, Exercise and Health Sciences, Loughborough University, Loughborough LE11 3TU, UK; stuart.cocksedge@coventry.ac.uk; 2 Centre for Physical Activity, Sport and Exercise Sciences, Coventry University, Coventry CV1 5FB, UK; 3 Fitoplancton Marino, S.L., Dársena Comercial s/n, 11500 El Puerto de Santa María, Cádiz, Spain; emg@easyalgae.com (L.M.); ecp@easyalgae.com (E.C.); carlos.infante@easyalgae.com (C.I.)

**Keywords:** *Tetraselmis chuii*, SOD, food supplement, cellular health, antioxidant, anti-inflammatory

## Abstract

*Tetraselmis chuii* (*T. chuii*) is a green, marine, eukaryotic, microalgae that was authorized in the European Union (EU) as a novel food for human consumption in 2014, and as a food supplement in 2017. This narrative review will provide an overview of preclinical and clinical trials assessing the efficacy of a *T. chuii*-derived ingredient, characterized by a high superoxide dismutase (SOD) activity (SOD-rich *T. chuii*), to improve various aspects of cellular health. Collectively, results from in vitro, and more importantly in vivo research, support SOD-rich *T. chuii* as a potential promoter of cellular health. Principally, the ingredient appears to function as an indirect antioxidant by boosting intracellular antioxidant systems. Moreover, it can positively modulate inflammatory status by up-regulating anti-inflammatory and down-regulating pro-inflammatory cytokines and factors. In addition, SOD-rich *T. chuii* appears to promote cellular health though protecting from DNA damage, boosting immune function, strengthening cell structure and integrity, and positively modulating cell signaling pathways. There is also some evidence to suggest that SOD-rich *T. chuii* may improve aspects of mitochondrial function through the up-regulation of genes linked to mitochondrial biogenesis and ATP synthesis. From the trials conducted to date, transcriptional activation of nuclear factor erythroid 2-related factor 2 (NRF2) and sirtuin 1 (SIRT1) appear to be important in mediating the effects of SOD-rich *T. chuii* on cellular health. These exciting preliminary observations suggest that SOD-rich *T. chuii* may represent a natural blue food supplement with the potential to enhance various aspects of cellular health.

## 1. Introduction

Microalgae are a wide group of single-celled photosynthetic organisms that represent the basis of aquatic food chains in both freshwater and seawater environments. From a phylogenetic standpoint, the term microalgae comprises both prokaryotic (cyanobacteria) and eukaryotic organisms, and it is considered to include 200,000–800,000 potential species [1]. One noteworthy feature of microalgae is their immense physiological plasticity, a characteristic that allows these organisms to survive in the most hostile environments, ranging from hypersaline waters to soils and rocks [2,3]. The capacity of microalgae to colonize such a diverse range of environmental conditions has been linked to their ability to produce primary and secondary metabolites with a highly diverse chemical nature (peptides and proteins, polyunsaturated fatty acids (PUFAs), polysaccharides, pigments, phytosterols, and/or phenolic compounds). These chemicals not only aid survival in microalgae, but are recognized to confer potential benefits for human health by exerting antidiabetic, antihypertensive, anti-obesity, anti-inflammatory, antimicrobial, or antiviral effects [4,5,6]. Consequently, there is increasing interest in the potential of consuming microalgae-containing supplements to enhance human health outcomes.

The marine eukaryotic microalgae *Tetraselmis chuii* (*T. chuii*) is a Chlorophyta included in the family *Chlorodendraceae*. A freeze-dried ingredient derived from this species is produced using a patent-protected technology under culture conditions that ensure a superoxide dismutase (SOD) activity above 30,000 U/g of the end product, which is significantly higher than that exhibited by other microalgae species [7,8]. In addition to high SOD activity, this SOD-rich *T. chuii*-derived ingredient (SOD-rich *T. chuii*) contains other compounds with potential bioactivity including PUFAs, vitamins, carotenoids, polyphenols, or phytosterols [9,10]. Initial work using pre-clinical experimental models has indicated that SOD-rich *T. chuii* can promote antioxidant and metal chelating responses, and inhibit acetylcholinesterase activities in in vitro tests, which could have positive implications for the prevention and/or management of neurological disorders, such as Alzheimer’s disease [11]. Moreover, immunostimulant effects of *T. chuii* were revealed in vivo in the gilthead seabream (*Sparus aurata*), as evidenced by increased hemolytic complement activity, phagocytic activity, serum levels of immunoglobulin M (IgM), expression of β-defensin, major histocompatibility complex II alpha chain (MHCIIα), and colony-stimulating factor-1 receptor (CSF1-R) in head kidney, as well as expression of occludin (a marker of the integrity of tight junctions) in the intestine [12,13,14]. Subsequent studies in preclinical mammalian animal models (rat) have reported indirect antioxidant properties of SOD-rich *T. chuii*, as well as its anti-inflammatory and immunostimulatory effects [15,16]. However, whilst these observations from preclinical studies were encouraging, clinical trials testing the bioactive effects of *T. chuii* and/or SOD-rich *T. chuii* in humans were lacking as such products had not yet been approved for human consumption.

Although *T. chuii* has been widely administered for fish, crustacean, and mollusk larvae feeding in the aquaculture industry [17], its use for humans has been limited by regulatory constraints. In 2014, *T. chuii* was authorized as a novel food for human consumption in the European Union (EU) according to Regulation (EC) No 258/97. Subsequently, in 2017, freeze-dried *T. chuii* was authorized as a food supplement in the EU [18]. These legislative changes opened new avenues for the application of SOD-rich *T. chuii*, including the possibility of assessing for potential health benefits of ingesting SOD-rich *T. chuii* in humans in vivo. This is important because, whilst SOD-rich *T. chuii* administration has been reported to enhance various aspects of cellular health in in vitro settings [19], the translation of such effects to humans could not be studied until recently and it is important to verify or refute the potential efficacy of SOD-rich *T. chuii* to promote human health. In this regard, clinical trials conducted in recent years have shown SOD-rich *T. chuii* to enhance physical performance and endogenous antioxidant responses in human skeletal muscle and to promote anti-inflammatory and immunostimulatory effects [16,20,21,22,23,24]. Importantly, these recent studies have shed light on the candidate molecular mechanisms underlying such physiological effects. Therefore, this narrative review aims to provide an overview of studies assessing the potential of SOD-rich *T. chuii* as a promoter of cellular health.

### Factors Contributing to Cellular Health

Cellular health broadly refers to the well-being and optimal functioning of individual cells. As cells aggregate to form tissues, which in turn form organs, and then organ systems that comprise the human body, good cellular health is crucial and foundational for overall health. Cellular health can be compromised by various endogenous and exogenous stressors (e.g., pollution, radiation, smoke, and/or oxidative stress). A myriad of factors influence cellular health, but this review will focus on redox balance, inflammation, immune and mitochondrial function, and cell structural integrity and signaling, which have the potential to be modulated by SOD-rich *T. chuii* supplementation.

Historically, redox state has been considered one of the most important factors affecting cellular health [25]. This redox state is determined by the balance between oxidants, such as reactive oxygen species (ROS) and antioxidants. Cells produce ROS as a consequence of metabolic processes. Organelles such as peroxisomes and mitochondria generate ROS as by-products, and different enzymes (xanthine oxidase, p450 cytochromes, and NADPH oxidase family) are well known to contribute to ROS production. While ROS have conventionally been considered cytotoxic derivatives of cellular metabolic processes, more recent work indicates that ROS production within a ‘physiological’ range is important for cellular redox signaling and health [26]. In healthy conditions, production of ROS is balanced by an elaborate defense system that includes both enzymes such as SOD, glutathione peroxidase (GPx), catalase (CAT), peroxiredoxins, and thioredoxins, and also non-enzymatic antioxidant scavengers such as vitamins C and E, and glutathione. However, when ROS production becomes excessive and ‘pathophysiological’, this can overwhelm the antioxidant defense systems, leading to a condition of oxidative stress, which reflects disruptive redox signaling and leads to the damaging of cellular constituents such as proteins, lipids, and DNA [27,28]. As such, maintaining an appropriate balance between ROS production and antioxidant response mechanisms is crucial to maintain cellular redox balance and health, and to prevent ill health and disease morbidity [25,29,30].

Inflammation is another important factor affecting cellular health. It is a fundamental part of immune function, being activated in response to microbial infections, tissue injury, or toxic compounds that disrupt tissue homeostasis [31]. Inducers of inflammation can be exogenous (pathogen-associated molecular patterns or virulence factors) or endogenous (signals derived from cells, tissues, plasma or extracellular matrix). These inflammatory responses are recognized by macrophages and mast cells, which initiate the production of inflammatory mediators, like chemokines and cytokines, with interleukin-1β (IL-1β), interleukin-6 (IL-6), and tumor necrosis factor-α (TNFα) being among the most important pro-inflammatory cytokines. Subsequent interaction of pro-inflammatory stimuli with their specific receptors triggers signaling pathways such as the mitogen-activated protein kinases (MAPK), and the nuclear factor kappa-B (NF-κB) pathway, which are known to play a key role in the immune response, survival, and apoptosis [32]. Although the inflammatory signaling cascade facilitates the elimination of the noxious stimuli and infection, it can also be harmful and damage healthy cells and tissues when the process is dysregulated [33]. This happens, for instance, during the so-called low-grade chronic inflammation, which has been associated with several age-related conditions, including the metabolic syndrome and cardiovascular disease [34]. To control inflammation, some immune cells produce anti-inflammatory cytokines such as interleukin-10 (IL-10), which inhibits the secretion of pro-inflammatory cytokines and promotes tissue repair [31,32].

The immune system is also recognized as being integral to the maintenance of optimal cellular health. In addition to providing defense against microorganisms, the immune system promotes elimination of damaged or abnormal cells that might degenerate in malignancies [35]. The effective execution of these functions is underpinned by an intricate network comprising physical barriers, like the skin; protein systems, like the complement and immunoglobulins; cellular elements, such as macrophages, lymphocytes, neutrophils, basophils, or eosinophils; surface receptors, like T- and B-cell receptors; and recognition molecules, like major histocompatibility complex I and II. Collectively, these act in host defense to restore tissue homeostasis and to protect and maintain health [36].

An additional factor contributing to cellular health is DNA integrity. Damage to DNA, which can be caused by ROS or radiation, can lead to mutations and contribute to premature aging and to several diseases, including cancer, if not properly repaired [25]. Moreover, it has been suggested that DNA mutations may alter transcriptional activity and alternative splicing of genes, which in turn might alter cellular homeostasis due to inadequate responses to endogenous or exogenous stressors [37,38].

Various aspects of mitochondrial function are key factors in determining cellular health. Principally, mitochondrial respiration is the most recognized aspect of mitochondrial function, during which the chemical energy-carrying molecule, ATP, is resynthesized via oxidative phosphorylation, and provides the necessary energy to fuel various essential cellular processes to preserve health and function. However, it is now clear that mitochondria are key orchestrators in a variety of additional key cellular processes, such as intracellular calcium signaling, proliferation, differentiation, and death or survival. Moreover, mitochondria contribute to the production of lipids, proteins, nucleic acids, and also neurotransmitters and hormones, and have an important role in iron homeostasis and the folate cycle [39,40]. This central role of mitochondrial function in cellular health is revealed by the number of diseases either underpinned or accompanied by dysfunction in one or more mitochondrial processes [41,42].

Two further factors with a significant effect on cellular health and are worthwhile mentioning within the context of the current review are cellular structural integrity and cell signaling. Regarding the structural integrity of the cell, eukaryotic cells exhibit a plasma membrane, acting as a barrier that separates the inside of the cell from the environment, and well-defined membrane-bound intracellular compartments. The membrane-bound intracellular compartments include a nucleus, endoplasmic reticulum, Golgi apparatus, and mitochondria, and whilst each compartment can orchestrate discrete cellular functions, mechanisms for inter-compartment communication pathways are recognized. Moreover, the dynamic structures belonging to the cytoskeleton (microtubules, actin filaments and intermediate filaments) are necessary to maintain cellular shape and internal organization, and are also important for supporting cellular processes such as cell division, cell movement, gene expression, or signal transduction. Maintaining this overall organization is crucial for cellular homeostasis and function [43,44,45]. Cellular health is also influenced by cell signaling, which allows cells to receive information from both the intracellular and extracellular stimuli and to initiate various effectors in response to such stimuli to preserve cellular and tissue homeostasis [46]. Amongst the effectors, intracellular signaling pathways that operate to convert intracellular or extracellular stimuli into cellular responses, MAPK cascades are one of the most conserved transduction pathways and play essential roles in key cellular processes such as division, motility, metabolism, apoptosis, differentiation, and stress responses [47].

There has been great interest in the health benefits that may be conferred by nutritional supplements with the potential to positively modulate cellular redox balance, inflammation, immune, and mitochondrial function, and cell structural integrity and signaling responses. The following sections will provide an overview of pre-clinical and clinical studies assessing the effects of SOD-rich *T. chuii* on these aspects of cellular health followed by a discussion of the results and a summary of the potential of SOD-rich *T. chuii* to improve cellular health.

## 2. Reported Studies Conducted with SOD-Rich *T. chuii* in Preclinical Models

In human skeletal muscle myoblasts, SOD-rich *T. chuii* has been reported to increase activity of the primary antioxidant enzymes (SOD, GPx, CAT) after 24 h of treatment, together with the transcriptional up-regulation of genes encoding such enzymes (SOD1, SOD2, GPx1, CAT) [19]. Moreover, this report was the first to demonstrate transcriptional activation of nuclear factor erythroid 2-related factor 2 (NRF2), a key transcription factor considered as the master regulator of the cellular antioxidant response, by SOD-rich *T. chuii* [48].

Results obtained in studies conducted with SOD-rich *T. chuii* using rodent models (rat) are summarized in Table 1. These studies can also be split into two completely different experimental designs. In three of these studies, animals consumed SOD-rich *T. chuii* (2.55 or 5.1 mg/Kg/day) for 6 weeks, and were subjected to a usual procedure for animal exercise training, including an initial week of adaptation to a treadmill system for the novel and stress impacts. Thereafter, animals ran on the treadmill five days per week at 25 m/min, 45 min/day, a model known to allow physical remodeling of the heart to increased oxygen consumption, and to improve contractile function and calcium handling of cardiac muscle [49]. These experiments were conducted in parallel with human trials (see Table 2), and were designed to serve as mechanistic models to investigate and help to understand the effects of SOD-rich *T. chuii* in physical performance and recovery, muscle strength and damage, and inflammatory and immunoregulatory responses [16,20,21]. Different markers were analyzed in rat muscle and serum samples, including antioxidant and oxidative stress-related parameters, markers of muscle tissue damage, myogenic factors, and pro-inflammatory cytokines.

The last of the trials was conducted to study the potential therapeutic effect of SOD-rich *T. chuii* in an animal model of metabolic syndrome, which is a cluster of metabolic disturbances such as abdominal obesity, hypertension, and hyperglycemia, and is characterized by low-grade chronic inflammation. Such dysregulations are known to represent a serious risk for developing cardiovascular disease and type 2 diabetes [50]. Metabolic syndrome was induced in animals by a diet rich in carbohydrates and fat, and low in fiber (cafeteria diet). Three different doses of SOD-rich *T. chuii* were evaluated (0.17, 1.7, and 17 mg/Kg/day), and a range of antioxidant, anti-inflammatory, and immune-modulatory markers were measured, including gene expression analysis in the liver, adipose tissue, thymus, and spleen [15].

## 3. An Overview of the Clinical Trials Conducted with SOD-Rich *T. chuii*

A summary of the most relevant data from clinical trials conducted with SOD-rich *T. chuii* in humans is presented in Table 2. In six of the reported studies, the effects of SOD-rich *T. chuii* supplementation on physical performance and related physiological parameters of healthy individuals were evaluated [16,20,21,22,23,51]. It is well known that the production of ROS increases in an intensity- and duration-dependent manner during skeletal muscle contractions, and although redox balance may be preserved by cellular antioxidant mechanisms, continued high rates of ROS production during intense or prolonged exercise can lead to exercise-induced oxidative stress [52]. Importantly, this exercise-induced oxidative stress has been linked to impairments in muscle perfusion and contractility, culminating in neuromuscular fatigue and impaired exercise performance [53]. In this scenario, it has been reported that dietary supplementation with some antioxidants has the potential to mitigate exercise-induced oxidative stress and to improve exercise performance, but such effects are inconsistent and require further investigation [54]. Given the potential for SOD-rich *T. chuii* supplementation to promote endogenous antioxidant enzyme responses, trials have been conducted in healthy human participants to examine its effects on various physiological and functional responses, as well as exercise performance and recovery [16,20,21,22,23,24]. In the only trial conducted to date to assess the effects in human skeletal muscle tissue directly via muscle biopsy sampling, gene expression changes in more than 100 key genes involved in antioxidant and inflammatory response were assessed using OpenArray™ technology (Thermo Fisher Scientific, Waltham, MA, USA). This work aimed to unravel some of the molecular basis underlying the potential ergogenic effects of SOD-rich *T. chuii* [24].

In addition to effects on exercise performance and recovery, two clinical trials have been conducted which investigated the effects of SOD-rich *T. chuii* supplementation in seminal parameters of idiopathic infertile men [51,55]. In this regard, oxidative stress is thought to be related to impairments in spermatogenesis, epididymal maturation, or sperm capacitation [56], and it has been negatively correlated with sperm count, motility, and morphology [57]. Whilst dietary consumption of antioxidants has been linked to improvements in sperm quality, outcomes are variable and appear to be depending on the ingredient investigated [58]. Thus, the potential effects of SOD-rich *T. chuii* as an indirect antioxidant on sperm quality were assessed in idiopathic infertile men. Participants consumed SOD-rich *T. chuii* for 90 days to cover a complete spermatogenesis cycle. Semen samples were collected for the determination of semen volume, as well as concentration, total number, progressive motility, and normal forms of sperm [51]. Subsequently, a second clinical trial has been conducted to confirm the previous outcomes in which additional relevant parameters related to sperm DNA integrity and seminal redox state have been investigated, and preliminary results have been already reported [55].

**Table 2:** Published studies reporting the effects of SOD-rich *T. chuii* in humans.

Trial Model	N	Dose (mg/day)	Duration	Main Outcomes	Reference
R, DB, PC, PM	18	25	14 days	↑ IMTP strength ↑ SJ power ↓ Serum CK activity	[16]
R, DB, PC, PM	32	25	30 days	↓ Heart rate ↑ $\dot{V}$O_2max_ ↑ Hemoglobin and MCH	[22]
R, DB, PC, PM	22	25	6 weeks	↑ PRS ↑ CMJ strength	[20]
R, DB, PC, PM	19	25	21 days	↑ IMTP force ↑ sIgA	[21]
R, DB, PC, PM	46	25/200	60 days	↑ Muscle percentage ↓ Fat percentage ↑ Basophils, monocytes, lymphocytes	[23]
R, DB, PM	40	25/250	90 days	↑ Semen volume ↑ Sperm concentration ↑ Total sperm number ↑ Sperm progressive motility	[51]
R, DB, PC, CO	13	25	14 days	↑ $\dot{V}$O_2_ peak Gene expression in muscle: ↑ NRF2, SIRT1, GPx7, PRDX6, PRDX3, c-JUN, MAPK14, GSR, GCLM, GSTM3, SOD2, CAT, CAPN3, AIFM1, CCL2, CASP8, IL-18, CUL3, BACH1 ↓ MAPK7, JUND	[24]
R, DB, PC, PM	80	250	90 days	↑ Total sperm number ↓ sORP ↓ DSBs	[55]

AIFM1: apoptosis inducing factor mitochondria associated 1; BACH1: BTB and CNC homology 1, basic leucine zipper transcription factor 1; BTBD1: BTB domain containing 1; CAPN3: calpain 3; CASP8: caspase-8; CAT: catalase; CCL2: C-C motif chemokine ligand 2; CK: creatine kinase; CMJ: counter movement jump; CO: cross-over; CUL3: cullin 3; DB: double blind; DSBs: double-strand DNA breaks; GCLM: glutamate-cysteine ligase modifier subunit; GPx7: glutathione peroxidase 7; GSR: glutathione-disulfide reductase; GSTM3: glutathione S-transferase mu 3; IMTP: isometric mid-thigh pull; IL-18: interleukin-18; MAPKP7: mitogen-activated protein kinase 7; MAPKP14: mitogen-activated protein kinase 14; MCH: mean corpuscular hemoglobin; N: number of participants; NRF2: nuclear factor erythroid 2-related factor 2; PC: placebo controlled; PM: parallel manner; PRDX3: peroxiredoxin 3; PRDX6: peroxiredoxin 6; PRS: perceived recovery status; R: randomized; sIgA: salivary immunoglobulin A; SIRT1: sirtuin 1; SJ: squat jump; SOD2: superoxide dismutase 2; sORP: static oxidation–reduction potential; *T. chuii*: *Tetraselmis chuii*. ↑ represents an increase in parameters or in expression of target genes, whereas ↓ represents a decrease in parameters or down-regulation of target genes.

## 4. Data Supporting SOD-Rich *T. chuii* as a Promoter of Cellular Health

**Figure 1:**
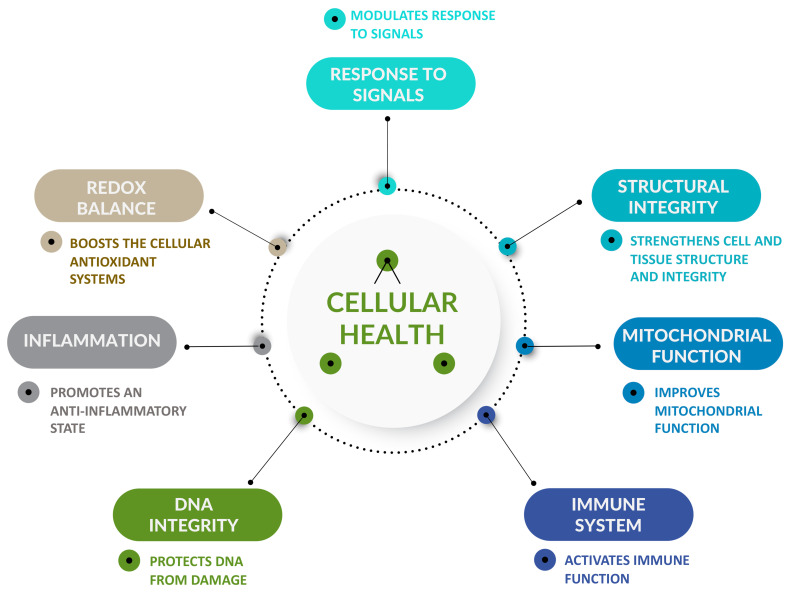
The effects of SOD-rich *T. chuii* on promoting cellular health.

When interpreted together, the outcomes from both preclinical and clinical trials previously shown collectively appear to support a role for SOD-rich *T. chuii* to positively promote some aspects of cellular health (Figure 1). Each of these points is substantiated in the sub-sections below.

### 4.1. SOD-Rich T. chuii Boosts the Cellular Antioxidant Systems

In a rodent model of metabolic syndrome, in which oxidative stress and low-grade chronic inflammation were induced by diet [15], supplementation with SOD-rich *T. chuii* reduced circulating levels of oxidized low-density lipoprotein (oxLDL). Serum oxLDL is derived from the reaction of LDL with peroxides or products generated from their degradation [59], and is considered a representative parameter of oxidative stress. As such, changes in plasma oxLDL in response to antioxidant-rich foods or dietary supplements are used to interpret their efficacy in improving redox balance [60,61,62]. Malondialdehyde (MDA) is another physiological parameter frequently measured as an indicator of oxidative stress [63,64,65]. MDA is probably the principal and most analyzed compound derived from lipid peroxidation, particularly from PUFAs, as they contain multiple carbon–carbon double bonds. In a physiological scenario of oxidative stress induced by physical exercise, SOD-rich *T. chuii* has demonstrated reduced MDA concentration, not only in serum but also in muscle tissue in rodent models, which supports the potential antioxidant effect afforded by SOD-rich *T. chuii* supplementation [16,21]. Another potential oxidative stress-related biomarker that has been reported to be attenuated after SOD-rich *T. chuii* consumption is the static oxidation–reduction potential (sORP) in seminal samples of idiopathic infertile men [55]. sORP is considered a direct measurement of oxidative stress or abnormal redox state in biological samples [66]. Particularly in semen samples, sORP has been demonstrated to provide very useful information about sperm function [67,68]. In aggregate, the improvement of these three oxidative stress parameters supports enhanced redox balance in cells after SOD-rich *T. chuii* administration.

Activation of the primary antioxidant enzymes (SOD, GPx, CAT) in response to SOD-rich *T. chuii* supports an improvement in the capacity of cells to restore and preserve redox homeostasis when encountering pro-oxidative insults. This concomitant activation of the three primary antioxidant enzymes has been observed in vitro in human skeletal muscle cells [19] and rat skeletal muscle tissue [16]. Moreover, an increase in the hepatic GPx activity after SOD-rich *T. chuii* consumption has been reported in a rodent model of metabolic syndrome [15]. These enzymes represent the first line of antioxidant defenses in the scavenging of free radicals in cells. SOD catalyzes the dismutation of superoxide radicals generated by different enzymatic systems in cells to hydrogen peroxide. Subsequently, GPx and CAT catalyze the reduction of hydrogen peroxide to oxygen and water, thereby converting a potentially pernicious molecule into stable byproducts [69]. In this context, activation of these primary antioxidant enzymes by dietary supplements with purported antioxidant effects is considered a much more effective therapeutic or complementary approach to combat cellular redox imbalance than supplementation with direct antioxidants [70,71,72]. The increase in the activities of these enzymes following SOD-rich *T. chuii* administration might be related to the transcriptional up-regulation of genes encoding such enzymes. In this regard, SOD-rich *T. chuii* increased the expression of SOD1 (the cytosolic Cu,Zn-SOD), SOD2 (the mitochondrial Mn-SOD), GPx1 (the most abundant selenoperoxidase, being virtually present in all cells) and CAT (mainly located at peroxisomes) genes in human muscle cells [19]. Similarly, an increase in transcripts of SOD1, SOD2, and GPx1 was detected in the liver of obese rats after SOD-rich *T. chuii* supplementation [15]. In human skeletal muscle, SOD2 and CAT genes were up-regulated by SOD-rich *T. chuii*, together with GPx7 [24]. Of note, GPx7 is a particular GPx that cannot bind glutathione, and hence, it has no capacity to participate in redox reactions directly. However, it is considered an essential sensor for oxidative stress and endoplasmic reticulum stress as it promotes signal transduction through interaction with additional factors [73].

SOD-rich *T. chuii* administration has been reported to increase the hepatic glutathione levels in a rodent model of metabolic syndrome [15]. Glutathione is a tripeptide comprising cysteine, glycine, and glutamic acid that can exist as a reduced form (GSH, or g-glutamyl–cysteinyl–glycine), which contains a thiol group on the cysteine residue and an oxidized form (GSSG), which corresponds to two glutathione molecules bound at the sulfur atoms via a disulfide bridge. Glutathione plays a crucial role in cellular antioxidant protection through direct chemical neutralization of free radicals (superoxide anion, hydroxyl radical, or nitric oxide), but it is also involved in the regeneration of oxidized vitamins C and E, and in the neutralization of reactive compounds such as peroxides or xenobiotics produced by detoxification enzymes. Moreover, it facilitates the plasma membrane transport of metabolites from toxins via formation of glutathione S-conjugates [74,75]. Glutathione biosynthesis occurs in the cytosol via two steps, both of which require ATP hydrolysis. The first one is the rate-limiting step and is catalyzed by the glutamate-cysteine ligase (GCL), in which g-glutamylcysteine is formed. The GCL comprises two subunits, a heavy catalytic subunit (GCLC), and a light regulatory or modifier subunit (GCLM) [76]. The second step is catalyzed by the glutathione synthetase (GSH-S), which adds glycine to form the final tripeptide. Another important enzyme is the glutathione-disulfide reductase (GSR), which catalyzes the reduction of GSSG to GSH [74,75]. The increase in liver glutathione content after SOD-rich *T. chuii* supplementation in obese rats might, therefore, be related to the up-regulated gene expression of GCLM, GSH-S, and GSR [15]. Moreover, SOD-rich *T. chuii* has been reported to increase the transcript levels of GCLM and GSR in human skeletal muscle of healthy subjects [24]. Collectively, these data support a role for SOD-rich *T. chuii* in the activation of de novo synthesis and recycling of GSH. This represents a valuable finding as dietary interventions that can directly stimulate GSH synthesis or prevent GSH depletion are of therapeutic interest [75].

Additional genes with an antioxidant function that are up-regulated after SOD-rich *T. chuii* supplementation in human skeletal muscle include peroxiredoxin 3 (PRDX3) and 6 (PRDX6) [24]. Peroxiredoxins are small non-selenoperoxidases found in all organisms that decompose hydrogen peroxide, lipid hydroperoxides, and peroxynitrite to form water and alcohols, thus protecting against oxidative damage. PRDX3 is restricted to the mitochondria, whereas PRDX6 is mainly located in the cytosol, and seems to play an important role in repairing oxidized cell membranes [77]. In addition, up-regulation of the glutathione-transferase mu 3 gene (GSTM3) in human skeletal muscle after SOD-rich *T. chuii* supplementation also is of value as GSTM3 serves as a phase II detoxification enzyme involved in maintaining redox homeostasis [78].

Regarding the underlying cell signaling that may contribute to the up-regulation of the aforementioned transcriptionally activated antioxidant genes by SOD-rich *T. chuii*, the Kelch-like ECH associated protein 1 (KEAP1)-NRF2-Antioxidant Response Element (ARE) is likely a key candidate signaling cascade. Indeed, KEAP1 interacts with NRF2 and acts as a sensor of cellular stress. It functions as an adaptor for the ubiquitin ligase complex, targeting NRF2 for ubiquitination and further degradation by the proteasome. Under oxidative conditions, KEAP1 undergoes a conformational change, which renders it unable to bind NRF2, and allows transport of NRF2 to the nucleus to activate transcription of target genes [79]. NRF2 belongs to the family of cap ‘n’ collar basic region leucine-zipper transcription factors, and binds to the AREs in the promoter region of target genes through heterodimerization with small musculoaponeurotic fibrosarcoma (Maf) proteins. Consequently, the NRF2 transcription factor is considered the master regulator of the antioxidant response in cells, and also controls the adaptive response to various environmental stressors. It is now recognized that NRF2 regulates the expression of more than 200 genes, most of them with a cytoprotective role, including genes involved in drug detoxification, lipid, and carbohydrate metabolism, as well as additional transcription factors [79,80]. In particular, NRF2 controls the expression of a fundamental set of genes involved in redox metabolism, like genes of the GSH-based system (e.g., GCLM, GCLC, or GSR), thioredoxin-based system (such as PRDX6), and other antioxidant systems such as SOD [79,80,81,82,83]. The pivotal role of NRF2 in cellular function is revealed by the complex regulatory mechanisms controlling its activity not only at transcriptional level but also at protein level, with polyubiquitination and further proteasomal degradation being one of the most important mechanisms [84]. Up-regulation of NRF2 by SOD-rich *T. chuii* has been demonstrated not only in vitro in human muscle cells [19], but, more importantly, in vivo in human skeletal muscle after a two-week supplementation period [24]. This represents a potential key finding in explaining the molecular mechanisms underlying the widespread stimulation of different cellular antioxidant systems by SOD-rich *T. chuii*, as outlined above, including up-regulation of both GCLM and GSR in rat liver and human skeletal muscle [15,24], SOD2 in human muscle cells and human skeletal muscle [19,24], or PRDX6 in human skeletal muscle [24]. Interestingly, another one of the known NRF2 target genes is GSTM3 [80], which is up-regulated by SOD-rich *T. chuii* in human skeletal muscle [24]. It has been reported that GSTM3 can prevent NFR2 polyubiquitination and further degradation by the proteasome, thus leading to activation and enhancement of NRF2 function [78].

In addition to NRF2, transcriptional activation of sirtuin 1 (SIRT1) by SOD-rich *T. chuii* in human skeletal muscle has also been observed. Sirtuins mediate the deacetylation of both histones and non-histone proteins in an NAD^+^-dependent manner, comprising a total of seven members in mammals, referred to as SIRT1 to SIRT7. Among them, SIRT1 is well known for its role in multiple biological processes, including cellular senescence, cell death, sugar and lipid metabolism, maintenance of genomic stability, inflammation, and also oxidative stress responses [85]. In this sense, it has been reported that SIRT1 can deacetylate NRF2, and this modification has been related to an increase in stability, nuclear localization, and transcriptional activity of NRF2 [86]. Thus, SIRT1 might improve cell resistance to oxidative stress-induced damage by increasing the expression of NRF2 and the downstream genes it activates with ARE [86,87,88]. Moreover, a positive regulation of SIRT1 by NRF2 at both protein expression and deacetylase activity has been reported, representing a positive feedback pathway in the cellular antioxidative response mediated by NRF2 [89]. As such, this interaction of SIRT1 with NRF2 could be considered as a mechanism to enhance the key antioxidant function of NRF2 and SOD-rich *T. chuii* might be mediating NRF2 signaling through SIRT1 up-regulation.

### 4.2. SOD-Rich T. chuii Promotes an Anti-Inflammatory State

Supplementation with SOD-rich *T. chuii* promoted an anti-inflammatory state in a rodent model of metabolic syndrome [22]. Indeed, one of the key findings was the transcriptional down-regulation of NF-κB1/p50 in the liver, thymus, and spleen of obesity-induced animals, restoring the transcript amounts to levels similar to those found in healthy animals. NF-κB1/p50 belongs to the family of inducible transcription factors involved in the inflammatory response. This family comprises four additional members, all of them being structurally related: NF-κB2/p52, RelA/p65, RelB, and c-Rel. All the members bind to the promoter region of the target genes in the form of homo- or heterodimers [90]. In normal conditions, family members are sequestered in the cytoplasm by inhibitory proteins like the IkB family members, with IkBa considered most important. Under several stimuli, a multi-subunit IkB kinase complex, or IKK, phosphorylates IkBa, triggering its degradation by the proteasome. This then allows the nuclear translocation of NF-κB members to the nucleus to mediate the transcriptional induction of pro-inflammatory cytokines in innate immune cells such as IL-1β or TNFα [91]. Thus, down-regulation of NF-κB1 after dietary supplementation with SOD-rich *T. chuii* might be related to the parallel reductions in IL-1β and TNFα transcripts in adipose tissue and thymus, or IL-1β in spleen [15]. Interestingly, SOD-rich *T. chuii* has also been demonstrated to reduce both serum and muscle levels of IL-1β and TNFα in response to intense exercise in a murine model [21], which supports more general anti-inflammatory protective properties of SOD-rich *T. chuii*. 

Significant down-regulation of the interferon γ (IFNγ) gene in adipose tissue, thymus, and spleen of dietary-induced obese animals has also been observed after supplementation with SOD-rich *T. chuii* [15]. IFNγ is the only characterized Type II interferon to date, and it is considered one of the most important cytokines mediating systemic and pathogenic inflammation in obesity [92,93]. In parallel to the IFNγ gene, SOD-rich *T. chuii* also elicited a significant down-regulation of the transforming growth factor-β1 (TGF-β1) gene in the liver. Significant and concomitant up-regulation of the IL-1β, TNFα, IFNγ, and TGF-β1 gene has been observed in the liver of high-fat fed mice, which were linked to the development of inflammation [94]. Additionally, transcriptional up-regulation of proinflammatory cytokines has been shown in primary human brain pericytes in response to TGF-β1 treatment [95]. Thus, down-regulation of the IFNγ and TGF-β1 genes in response to SOD-rich *T. chuii* might contribute to the anti-inflammatory effect of the ingredient.

Interestingly, dietary supplementation with SOD-rich *T. chuii* positively regulated the pleiotropic anti-inflammatory cytokine IL-10 in a rodent model of metabolic syndrome [15]. In this regard, serum levels of IL-10 in supplemented animals were restored to similar values exhibited by healthy animals. In parallel, the IL-10 gene was up-regulated in adipose tissue, and to a higher degree in immune organs such as the thymus and spleen. It has been shown that IL-10 inhibits, at the transcriptional level, the expression of the pro-inflammatory cytokines IL-1β and TNFα via the blockade of NF-κB nuclear localization [96,97], which agrees with the down-regulation of both cytokines induced by SOD-rich *T. chuii*, as previously mentioned.

In adipose tissue of obese rats, SOD-rich *T. chuii* significantly increased adiponectin (ACDC) transcripts [15]. ACDC is an adipokine with anti-diabetic, anti-atherogenic, and anti-inflammatory effects, which is expressed mainly in adipose tissue but also in a variety of different tissues like myocytes or epithelial cells [98]. In obese mice, ACDC has been shown a negative effect on IFNγ production by CD4+ T cells [99]. Moreover, it has been reported that ACDC can induce the production of anti-inflammatory IL-10 in macrophages and dendritic cells, while concomitantly suppressing the production of proinflammatory TNFα and IFNγ in stimulated macrophages [100]. Therefore, the existing data suggest that SOD-rich *T. chuii* can modulate the complex and interconnected network of cytokines and factors controlling the inflammatory response to promote anti-inflammatory effects in a variety of tissues and organs.

The transcriptional activation of both NRF2 and SIRT1 observed in human skeletal muscle following SOD-rich *T. chuii* supplementation might also play a key role in its anti-inflammatory effects. In this regard, it has been shown that a fine-tuning regulatory mechanism operates between NRF2 and NF-κB, with NRF2 negatively regulating the pro-inflammatory NF-κB signaling pathway via different pathways [101,102]. For instance, as a consequence of NRF2 activation, cellular antioxidant defenses are increased, which reduces ROS bioavailability and thus, activation of the IKK complex and further phosphorylation of IkBa, thereby inhibiting nuclear translocation of NF-κB members to the nucleus. In this way, transcriptional induction of pro-inflammatory cytokines is down-regulated. Moreover, it has been shown that NRF2 activation can prevent transcriptional up-regulation of pro-inflammatory cytokines, including IL-1β or IL-6, by directly binding to the promoter region of these genes, thus inhibiting recruitment of RNA polymerase II and transcription initiation [103]. Considering SIRT1 expression is increased following SOD-rich *T. chuii* supplementation, alternative mechanisms might contribute to the anti-inflammatory effects elicited by SOD-rich *T. chuii* [104]. For instance, SIRT1 is known to directly deacetylate RelA/p65, one of the NF-κB subunits, thus inhibiting pro-inflammatory cytokine expression mediated by NF-κB [105]. Moreover, this deacetylation has been related to an enhancement of RelA/p65 methylation, which leads to an increase in RelA/p65 ubiquitination and further degradation by the proteasome, and hence, inhibition of NF-κB transcriptional activity [106]. SIRT1 can also negatively affect DNA binding of NF-κB subunits through accumulation in the promoter region of pro-inflammatory cytokines [107]. Additionally, it has been reported that SIRT1 can inhibit IkB degradation, thus avoiding nuclear accumulation of NF-κB components and down-regulating NF-κB function [108].

### 4.3. SOD-Rich T. chuii Protects DNA from Damage

In a clinical trial (Table 2), preliminary data analysis has demonstrated that SOD-rich *T. chuii* lowered double-strand DNA breaks (DSBs) in the sperm of idiopathic infertile men after 90 days of supplementation [55]. This may have important implications for sperm quality as high levels of DSBs have been related to a diminished chance of conceiving and to a higher incidence of miscarriage when fathering a pregnancy [109]. Although less frequent than single-strand DNA breaks (SSBs), DSBs are considered particularly harmful lesions as they provoke genome instability and chromosomal rearrangements. They can originate because of under replicated DNA during cell division, and also by the action of transposable elements or ionizing radiation. This ionizing radiation breaks water molecules to create hydroxyl free radicals, which can react with DNA to produce SSBs and these SSBs can be spontaneously converted in DSBs directly [110]. Thus, and as previously addressed, the induction of the cellular antioxidant mechanisms and the corresponding increase in the scavenging capacity of free radicals mediated by SOD-rich *T. chuii* might be related to the decrease in DSBs. Indeed, antioxidants have been demonstrated to protect against DSBs [111,112,113,114].

Reduction of DSBs might also be related to an improvement in the activity of DNA repair mechanisms. The two main methods involved in DSB repair are homologous recombination (HR), in which sister chromatids serve as templates during the process of repair, and nonhomologous end joining (NHEJ). In both instances, a complex molecular machinery involved first in the recognition of DSBs and then in DNA synthesis and repair is known to operate [115]. In this scenario, up-regulation of SIRT1 mediated by SOD-rich *T. chuii* might have a role in decreasing DSBs as SIRT1 activity has been strongly related to repair of DSBs [116]. For instance, SIRT1 deacetylates the repair factor Ku70, enhancing DNA repair capacity [117], and maintains the acetylation level of nibrin (NBS1), which is a component of a conserved nuclease complex that acts as a critical sensor in regulating cellular responses to DSBs for efficient DNA damage repair [118]. Moreover, SIRT1 is also involved in the selection of the DSB repair pathway via deacetylation of the KRAB-associated protein 1 (KAP1) to promote NHEJ, suppressing the HR repair pathway [119].

The potential contribution of NRF2 activation by SOD-rich *T. chuii* in the reduction of DSBs may also be of value regarding DNA repair. In this sense, several DNA repair genes involved in the HR pathway are likely to be regulated by NRF2 as they exhibit AREs in their promoter regions [120]. Moreover, NRF2 can activate the ataxia telangiectasia mutated (ATM), which is a master regulator of the DNA damage response, leading to G2 cell cycle arrest and promoting the HR repair of DSBs to preserve genome stability [121]. In addition, NRF2 seems to be also involved in NHEJ [122].

### 4.4. SOD-Rich T. chuii Activates Immune Function

Supplementation with SOD-rich *T. chuii* for three weeks has been shown to sustain immune function in individuals after an intensified resistance training protocol, as revealed by better maintenance of salivary immunoglobulin A (sIgA), compared to a placebo group [21]. The sIgA molecule as a secretory IgA is formed by a dimeric IgA and a glycoprotein known as the secretory component, stabilizing and protecting the molecule from degradation by bacterial and digestive enzymes. It is known that sIgA can prevent bacterial colony formation on mucosal surfaces, and can also neutralize toxins and enzymes produced by bacteria, and also pathogenic viruses inhibiting penetration into epithelial cells [123]. Regarding athlete illness, sIgA has been used as a valuable biomarker to evaluate the risk of developing respiratory tract infections [124]. Indeed, a decrease in sIgA levels after a long duration and high-intensity exercise seems to be associated with increased upper respiratory symptoms [124,125]. For instance, college football players exhibited a reduction in sIgA concentration and an increase in the incidence of upper respiratory tract infections over 12 months [126]. Conversely, a decrease in respiratory symptoms was observed in individuals after 12 weeks of moderate exercise training, with a parallel increase in the concentration of sIgA [127]. Thus, a possible contribution of SOD-rich *T. chuii* to improving immune function is inferred from better sIgA during intensified exercise training.

An increase in counts of different white cells such as basophils, monocytes, and particularly, lymphocytes, has also been observed after supplementation with SOD-rich *T. chuii* [23]. Other dietary interventions have also been shown to increase lymphocyte count. Indeed, an association between an increase in lymphocyte numbers and the improvement in the immune status of individuals has been reported after supplementation with different antioxidants, such as vitamins, b-carotene, and selenium [128,129,130], and also with the microalgae Spirulina [131]. The presence of acidic and sulfated polysaccharides in the cell wall of the microalgae *Tetraselmis* has been suggested as a potential inductor of immune cell proliferation through the increase in cytokine and chemokine production by macrophages [23].

Transcriptionally activated genes by SOD-rich *T. chuii* in human skeletal muscle are known to play a role in immune function [24], specifically, the monocyte chemoattractant protein-1 (MCP-1)/CC chemokine ligand-2 (CCL2). Chemokines are small (8–14 kDa) signaling proteins d, which are secreted by different immune cells. Chemokines comprise four families with two main subgroups (CXC and CC) and two small subgroups (CX3C and C), with MCP-1/CCL2 belonging to the CC family. MCP-1/CCL2 protein is mainly produced by epithelial cells, endothelial cells, smooth muscle cells, monocytes/macrophages, fibroblasts, astrocytes, and microglial cells, which are regulated by several other cytokines and factors. Importantly, it is known that MCP-1/CCL2 can direct the migration and infiltration of monocytes at the site of injury and infection, and are also involved in proliferation of T cells, thus important to the immune response [132]. Interleukin-18 (IL-18) is another of the genes up-regulated by SOD-rich *T. chuii*. It belongs to the IL-1 family of cytokines, and is a potent pro-inflammatory cytokine involved in host defense against infections through innate and acquired immune stimulation responses. IL-18 is produced by hematopoietic cells (such as monocytes and macrophages) and non-hematopoietic cells (for instance, keratinocytes and mesenchymal cells). Together with IL-12, IL-18 triggers the innate immune system, stimulating NK cells to respond to cancer and infections, as well as to activate macrophages. In the adaptive immune system, IL-18 promotes the activation and differentiation of T cells and is essential for the development of natural killer (NK) cells, and also up-regulates the cytotoxic activities of NK and CD8+ T cells [133]. Interestingly, IL-18, as occurs with other interleukins of the same family, is synthesized in the cytoplasm as an inactive precursor referred to as pro-IL-18. This precursor is further transformed into the active IL-18 in multiprotein cytosolic complexes named inflammasomes in a caspase-1 (CASP1)-mediated process. However, an additional activated gene by SOD-rich *T. chuii* is caspase-8 (CASP8), which has also been involved in the processing of inactive IL-18 into the active form in a CASP1-independent process [134].

Transcriptional up-regulation of NRF2 by SOD-rich *T. chuii* [24] might also have a direct influence in the regulation of immune function based on the known roles of this transcription factor in immunity [79,135]. For instance, NRF2 is involved in bacterial clearance via up-regulation of the macrophage receptor with collagenous structure (MARCO) gene, which encodes a scavenger receptor necessary for bacterial phagocytosis [136]. An antitumor and antiviral role has also been observed for NRF2 via activation of the cytokine IL-17D, which exhibits tumor rejection activity mediated by NK cells [137]. Moreover, a role of NRF2 in T-cell differentiation has been observed, favoring Th2 but decreasing Th17 [138,139]. On the other hand, SIRT1 has also been implicated in the immune response, and hence up-regulation of SIRT1 gene expression could contribute to improved immune responses after SOD-rich *T. chuii* supplementation [24]. For instance, regarding innate immunity, SIRT1 influences myeloid-derived suppressor cells (MDSCs) differentiation, and regulates the generation of cytokines by dendritic cells, subsequently modulating their function. In the adaptive immune response, SIRT1 can influence the differentiation of inflammatory T cells, and plays an essential role in Th17 formation and in the activation of B cells, facilitating immune function [140,141].

### 4.5. SOD-Rich T. chuii Potentially Improves Mitochondrial Function

Although there is currently no direct experimental evidence of SOD-rich *T. chuii* modifying key parameters of mitochondrial physiology, data from clinical trials suggest a potential positive effect of SOD-rich *T. chuii* on aspects of mitochondrial function. Positive regulation of SIRT1 by SOD-rich *T. chuii* [24] might be involved in increasing mitochondrial content as SIRT1 is a well-known promoter of mitochondrial biogenesis through the activation, by deacetylation, of the peroxisome proliferator-activated receptor γ-coactivator-1α (PGC-1α). In turn, activated PGC-1α activates the mitochondrial transcription factor A (TFAM) in the cytoplasm, eliciting the import of both SIRT1 and PGC-1α into the mitochondria and the recruitment of TFAM to the D-loop region of mitochondrial DNA, where it forms a multiprotein complex with SIRT1 and PGC-1α. Finally, this complex drives the replication and transcription of mitochondrial DNA to improve mitochondrial biogenesis [142]. In addition, up-regulation of NRF2 induced by SOD-rich *T. chuii* [24] may also have contributed to mitochondrial biogenesis. Indeed, NRF2 controls the expression of the nuclear respiratory factor 1 (Nrf-1), which in turn activates TFAM, leading to mitochondrial DNA replication. NRF2 can also contribute to mitochondrial function through the induction of mitophagy, a process in which damaged mitochondria are removed from the cell, and for its key role in maintaining mitochondrial membrane potential, which increases the efficiency of oxidative phosphorylation and ATP production [143,144]. Another gene known to play a key homeostatic role in mitochondrial function is the apoptosis-inducing factor mitochondria associated 1 (AIFM1), which was also found to be up-regulated by SOD-rich *T. chuii* in skeletal muscle [24]. This gene encodes a mitochondrial oxidoreductase that takes part in the electron chain assembly, and thus regulates oxidative phosphorylation and ATP production [145].

Up-regulation of genes encoding the key mitochondrial antioxidant proteins SOD2 and PRDX3 [24] by SOD-rich *T. chuii* might also contribute to improved mitochondrial function as they act to protect the organelle from damage caused by ROS. In this sense, a role for the NRF2 pathway in maintaining mitochondrial homeostasis through the activation of antioxidant and quality control genes has been stated [146].

The MAPK signaling pathway has also been involved in mitochondrial physiology. In this regard, activation of MAPK/p38 signaling has been shown to enhance PGC-1α levels and activity, thus promoting mitochondrial biogenesis [147,148]. Indeed, MAPK/p38 has been shown to phosphorylate PGC-1α in muscle cells directly, enhancing its activity via the increase in protein stability and through the inhibition of the interaction with its co-repressor [149]. Moreover, MAPK/p38 signaling is currently known to regulate the activity of key mitochondrial proteins involved in oxidative phosphorylation and iron homeostasis [150]. In this scenario, up-regulation of MAPK14/p38α by SOD-rich *T. chuii* might be an additional pathway contributing to the improvement of mitochondrial function.

### 4.6. SOD-Rich T. chuii Strengthens Cell and Tissue Structure and Integrity

Supplementation with SOD-rich *T. chuii* has been related to a decrease in markers of cellular and tissue damage, which suggests a strengthening effect on cellular structure. In this regard, serum creatine kinase (CK) and myoglobin were significantly reduced after a cross-training event in endurance-trained individuals and mechanistic rodent models after SOD-rich *T. chuii* supplementation [16,20]. Serum content of skeletal muscle enzymes (such as CK) and proteins (such as myoglobin) are markers of the functional status of muscle tissue, and can vary widely depending on physiological conditions. An increase in CK is considered an index of tissue damage following acute and chronic muscle injuries, as it is known that strenuous exercise, particularly incorporating eccentric contractions, can damage skeletal muscle cell structure, which increases CK efflux into the systemic circulation [151,152]. Myoglobin, a monomer protein involved in oxygen storage, is released to serum as a result of degradation of muscle cells following strenuous exercise [151,153]. These results might be related, at least in part due to the indirect antioxidant effects mediated by the ingredient helping to mitigate the harmful effects caused by exercise-induced oxidative stress, thus reducing protein damage (e.g., carbonylation and cross-linking) and further degradation by the proteasome, and also damage to cellular membranes related to lipid peroxidation [154]. Moreover, in a mechanistic rodent model, SOD-rich *T. chuii* has been shown to increase the muscle protein content of the muscle atrophy F-box (MAFbx) and muscle RING-finger protein-1 (MuRF-1) [20], two ubiquitin ligases involved in protein degradation by the proteasome [155], which might be related to an increase in protein turnover to counteract protein breakdown linked to exercise and oxidative stress [20].

Some well-known markers linked to tissue repair and regeneration have been shown to be activated by SOD-rich *T. chuii*. Indeed, an increase in the muscle protein content of the myogenic differentiation factor, MyoD, and neural cell adhesion molecule, NCAM, was detected in response to SOD-rich *T. chuii* in a rodent model [20]. Both proteins are key positive regulators of satellite cell progression [156,157], which are mononuclear dormant cells that are activated to promote regeneration upon fiber damage. After muscle injury, these satellite cells are activated and differentiate to myoblasts, which exit the cell cycle and become myocytes after several rounds of proliferation. Ultimately, myocytes undergo a fusion process to form multinucleated myotubes that eventually mature into myofibers [158]. In contrast and in the same mechanistic model, SOD-rich *T. chuii* reduced levels of myostatin [20], a paracrine signaling molecule mainly secreted by skeletal myocytes that behaves as a negative regulator of muscle growth and differentiation [159].

In human skeletal muscle, SOD-rich *T. chuii* transcriptionally activated different genes with myogenic functions [24], thereby representing an additional support role for SOD-rich *T. chuii* in the processes of repair and regeneration of tissues. For instance, BTBD1, a gene that encodes a BTB (broad-complex, tramtrack, and bric-a-brac) domain and facilitates interaction with other proteins lacking this domain, is known to interact with DNA topoisomerase I, and is expressed in many human tissues, with higher levels in heart and skeletal muscle [160]. Interestingly, BTBD1 has been demonstrated to be essential for myogenic differentiation [161]. In addition, calpain 3 (CAPN3) gene expression was also up-regulated by SOD-rich *T. chuii*. Calpains are a family of Ca^2+^-dependent cysteine proteases that participate in various cellular processes. In particular, CAPN3 is the only muscle-specific calpain, and has important roles in the promotion of calcium release from skeletal muscle fibers, calcium uptake of sarcoplasmic reticulum, and also in muscle formation and remodeling [162]. Therefore, as a potential contribution of CASP8 and/or caspase-10 (CASP10), both genes also up-regulated in response to SOD-rich *T. chuii* treatment, and might also be considered to aid tissue structure and integrity. Caspases are a family of cysteine-aspartate proteases involved in cell death processes [163], but it is currently known that some of these proteins like caspase-2 (CASP2) and caspase-3 (CASP3) can play a direct role in skeletal muscle differentiation and myogenesis [164,165], allowing the muscle remodeling required for differentiation but being inhibited before their activation can lead to cell death. Finally, given the role of NRF2 in the stimulation of muscle repair, growth, and differentiation [166], up-regulation of this key transcription factor by SOD-rich *T. chuii* might also contribute to improved muscle regeneration and integrity.

### 4.7. SOD-Rich T. chuii Modulates Cellular Signaling

A role for SOD-rich *T. chuii* in the modulation of cellular signaling is supported by the modification, in human skeletal muscle tissue, of transcript amounts of up to four different genes of the MAPK signaling pathway: MAPK1/ERK2, MAPK6/ERK3, MAPK7/ERK5, and MAPK14/p38α [24]. The three conventional MAPKs (MAPK1/ERK2, MAPK7/ERK5, and MAP14/p38a) were up-regulated, and the atypical MAPK6/ERK3 was down-regulated by SOD-rich *T. chuii*. In eukaryotic cells, involvement of the MAPK pathway in key physiological processes (mitosis, metabolism, motility, survival, apoptosis, differentiation, and response to stress) is well established and works in four different signaling cascades: the extracellular signal-regulated kinases 1/2 (ERK1/2), c-Jun amino (N)-terminal kinases 1/2/3 (JNK1/2/3), p38 isoforms (α, β, γ, and δ), and ERK5 [167]. In a general view, different studies suggest that the MAPK/ERK signaling pathway is closely related to processes such as cellular proliferation and differentiation, whereas the JNK and p38 pathways seem to be more related to response to stress and apoptosis [47].

At the cellular level, the actual physiological significance of the modified expression of MAPK genes is currently unknown, but the cross-talk between MAPKs and key factors and metabolic processes are worthy of discussion. For instance, the MAPKs signaling cascade can be activated by ROS, which has been associated with NRF2 function [168]. In this regard, it is known that MAPK1/ERK2 can directly phosphorylate NRF2, which positively regulates NRF2 activity. Moreover, it has been demonstrated that MAPK14/p38α can phosphorylate NRF2, although both positive and negative regulation of NRF2 activity has been reported. Irrespective, it seems that phosphorylation of NRF2 by MAPKs only slightly affects NRF2 transactivation and further expression of NRF2 target genes, suggesting that MAPKs might regulate NRF2 activity mainly in an indirect manner [169]. In this regard, an additional point of relevance is the fact that phosphorylation and nuclear accumulation of MAP14/p38a can be stimulated by SIRT1, which has been related to the promotion of cell proliferation [170].

The activator protein-1 (AP-1) is also relevant regarding MAPK cross-talk. AP-1 is a transcription factor that consists of different components, such as the Jun family (which includes c-JUN and JUND), Fos family, Jun-dimerizing partners (JDP), musculoaponeurotic fibrosarcomas (Maf) family, and activating transcription factor (ATF) family. Several stimuli, including oxidative stress and intracellular MAPK signaling, can activate AP-1, mediating functions such as cell growth and differentiation [171]. In human skeletal muscle, SOD-rich *T. chuii* has been shown to up-regulate both c-JUN and ATF1 and to down-regulate JUND [24]. Understanding the physiological relevance of such modifications in gene expression will require further research, but the existence of a complex fine-tuned regulatory network modulated by SOD-rich *T. chuii* to maintain an adequate physiological balance in the cell is suggested. Indeed, JUND can activate NRF2-induced transcription of downstream genes, with MAPK signaling playing a central role in this process [172], with c-JUN, a known target of SIRT1, inhibiting AP-1 transcriptional activity [173]. It should also be mentioned that AP-1 mediates the expression of inflammatory mediators, such as cyclooxygenase 2 and prostaglandin E2 [141], and hence the inhibition of AP-1 signaling by SIRT1 potentially represents an additional anti-inflammatory mechanism of SOD-rich *T. chuii* which may be mediated through SIRT1 up-regulation.

### 4.8. SOD-Rich T. chuii Induces a Homeostatic Response

In human skeletal muscle, a deeper gene expression analysis revealed a potentially interesting series of observations. In more than half of the genes analyzed using the OpenArray™ technology, a negative correlation was found between the baseline gene expression and the magnitude of increase in transcript amounts after supplementation with SOD-rich *T. chuii* for two weeks [24]. This suggests that individuals with the lowest expression levels exhibited the highest responsiveness to SOD-rich *T. chuii* supplementation. As such, SOD-rich *T. chuii* seems to act as a modulator of cellular responses that need enhancement. This finding is of potential value as both direct and indirect antioxidant supplements have been used to offset the potentially harmful effects of excess free radicals. However, it has more recently been considered that physiological levels of such free radicals control fundamental biological processes [174], and excess antioxidants could interfere with beneficial redox-mediated cellular signaling [26]. Consistent with this interpretation, it has been shown that over-stimulation of the NRF2/SIRT1 pathway, which regulates the activity of the cellular antioxidant and anti-inflammatory mechanisms, can lead to deleterious outcomes, even causing reductive stress and adverse health effects [175,176,177]. Instead, the existing data suggest that SOD-rich *T. chuii* may behave as a transcriptional activator of this pathway in a controlled manner, as supported by concomitant improvement in various physiological responses, in both preclinical models and clinical trials.

As observed in human skeletal muscle, transcriptional activation of additional genes, such as cullin 3 (CUL3) and BTB and CNC homology 1, basic leucine zipper transcription factor 1 (BACH1) by SOD-rich *T. chuii* might somehow elicit a balanced and controlled cellular response to stress mediated by the NRF2/SIRT1 pathway [24]. Indeed, CUL3 is a protein known to behave as a negative regulator of NRF2 function through mediating its ubiquitination and proteasomal degradation [84]. BACH1 is a transcription repressor that is conserved and ubiquitously expressed in tissues. In the absence of cellular stress, BACH1 forms heterodimers in the nucleus with small Maf proteins that bind to the AREs and repress defensive gene expression mediated by NRF2. Thus, BACH1 acts as a negative regulator of NRF2 nuclear levels and function [178]. Therefore, increased CUL3 and BACH1 gene expression after SOD-rich *T. chuii* might modulate and fine-tune NRF2 expression and its subsequent effects to promote a healthy adaptive response, as opposed to excessive and maladaptive responses.

### 4.9. Summary of Data Supporting SOD-Rich T. chuii as a Promoter of Cellular Health

Considering the existing empirical data, SOD-rich *T. chuii* may be considered as a potential cellular health promoter. This statement is based on the ability of SOD-rich *T. chuii* to (i) boost the cellular antioxidant systems to help combat the harmful effects of oxidative stress; (ii) promote an anti-inflammatory state through activating anti-inflammatory and down-regulating pro-inflammatory factors; (iii) protect DNA damage by reducing double-strand DNA breaks and up-regulating key genes involved in maintaining genome stability; (iv) improve immune function by up-regulating genes known to be involved in the host defense against pathogens, increasing levels of immunoglobulins and counts of different immune cells; (v) potentially improve mitochondrial function by up-regulating pivotal genes involved in mitochondrial biogenesis and ATP production, as well as genes coding for mitochondrial markers involved in the protection against free radicals; (vi) strengthen cell and tissue structure and integrity through reducing markers of cellular and tissue damage, and up-regulating genes involved in the protection and repair of membranes, and promoting cellular differentiation and tissue regeneration; (vii) modulate cellular signaling by enhancing the expression of key genes that participate in signaling pathways. Two key factors transcriptionally up-regulated by SOD-rich *T. chuii*, NRF2 and SIRT1, appear as central players that might mediate the cellular health effects of the ingredient as they are themselves considered as promoters of cellular homeostasis and health [179,180].

## 5. Prospects

Despite encouraging initial evidence to support improved cellular health and homeostasis after SOD-rich *T. chuii* supplementation, it is recognized that further research is necessary to unravel the full molecular mechanisms and pathways that support the health effects SOD-rich *T. chuii*. In this regard, some key mediators, like NRF2 and SIRT1, which are well-known pivotal factors for cellular health and homeostasis, have been identified and are worthy of further empirical investigation. Indeed, NRF2 and SIRT1 genes are up-regulated after SOD-rich *T. chuii* supplementation [24]. However, transcriptional control is not the only regulatory level of gene function acting on cells. Particularly for NRF2, its activity is affected by post-translational modifications such as ubiquitination, sumoylation, acetylation, or phosphorylation, which control protein degradation by the proteasome and its nuclear accumulation and stability [84]. SIRT1 activity is also modulated by post-translational modifications such as ubiquitination, sumoylation, phosphorylation, and methylation, which regulate protein degradation, stability, and affinity toward its target proteins [142]. Thus, future attempts to identify all the regulatory mechanisms modulated by SOD-rich *T. chuii*, particularly concerning NRF2 and SIRT1, will undoubtedly help to better understand the molecular bases of the physiological benefits it may confer.

As presented in this review, the clinical trials conducted to assess the potential beneficial health effects of SOD-rich *T. chuii* have been performed in two particular and quite different physiological scenarios, including sports nutrition and male infertility. By taking into account the molecular mechanisms supporting SOD-rich *T. chuii* as a promoter of cellular homeostasis and health, particularly with the transcriptional activation of NRF2 and SIRT1, additional health applications become of potential interest to explore with the supplement. For example, a novel therapeutic use of SOD-rich *T. chuii* could be useful to combat age-related health declines. Aging is characterized by a decline in various functional responses owing to the accumulation of cellular damage. Several factors have been identified as key contributors to the aging process, including, among others, genome instability, loss of proteostasis, mitochondrial dysfunction, and chronic inflammation [181,182]. It has been shown that NRF2 transcriptional activity decreases with age [183], and this gradual reduction of NRF2 is considered to drive aging owing to increased oxidative stress, which contributes to various hallmarks of aging [184]. In this scenario, the induction and activation of the NRF2 pathway to maintain cellular antioxidant function and redox balance has been suggested as a targeted therapeutic strategy to reduce cell and tissue damage, which is known to occur in age-related ocular [185], joint [186], skin [187], kidney [188], liver [189], cardiovascular [190], and neurodegenerative [191] disorders, and could, therefore, help ameliorate symptoms and offset disease morbidity and progression. In addition to NRF2, it is known that SIRT1 exhibits an age-dependent decrease in expression both at protein and transcription levels [192], such that activation of SIRT1 has also been proposed as an effective means to improve age-related disorders [193,194,195,196]. Hence, in this scenario, the potential contribution of SOD-rich *T. chuii* to the improvement of age-related disorders and diseases through the activation of NRF2 and SIRT1 becomes of interest, although specific clinical trials need to be conducted to evaluate its efficacy in this regard.

A potential therapeutic use of SOD-rich *T. chuii* for women’s health through the improvement of cellular function is also of potential interest. It is known that oxidative stress and inflammation are important negative factors in several aspects of women’s reproductive physiology, and hence, SOD-rich *T. chuii* might help to restore the redox balance and to promote an anti-inflammatory state in target cells. For instance, it is known that ROS negatively affect the maturation and development of the oocyte, implantation and luteolysis, and hence, a favorable redox balance appears to be crucial for the oocyte maturation and quality, and also for placentation, fetal growth, and organ development. Moreover, oxidative stress and inflammation mediates the acceleration of pathology in the female reproductive tract, including primary ovarian insufficiency, polycystic ovary syndrome, endometriosis, endometrial hyperplasia, and preeclampsia [197]. In this scenario, interventions to reduce the impact of ROS, and hence, the quality of embryos and implantation, are considered an adequate strategy for a successful pregnancy. Thus, it has been stated that the use of supplements to activate the NRF2/SIRT1 pathway and hence to improve antioxidant and anti-inflammatory activities in female reproductive organs represents a fruitful approach in treating female reproductive disorders [198,199,200,201]. In addition, it has been reported that postmenopausal women are at a high risk for oxidative stress due to a marked reduction in estrogen production. The increase in the serum concentration of inflammatory cytokines, together with the increase in pro-oxidant biomarkers and the decrease in antioxidant biomarkers are among the main features related to the menopausal transition [202,203]. Thus, the elevation of cytokines and pro-oxidant markers suggests that there is a high degree of oxidative stress in the postmenopausal state. All these features are mainly due to estrogen deprivation, and hence, it might be linked with the development of postmenopause-associated increased cardiovascular risk, bone density loss in osteoporosis, hot flushes, oral dryness, loss of muscle mass and strength, atherosclerosis, weight gain, or disorders such as depression and anxiety [204,205,206,207,208]. In this scenario, a large body of evidence suggests that the NRF2/SIRT1 pathway is involved in protection against the physiological impairments that develop observed in postmenopausal women. Indeed, it is known that estrogens activate NRF2 and SIRT1, and the decrease in estrogen production during menopause has been related to a range of pathologies related to oxidative stress and inflammation including, among others, osteoporosis, cardiovascular diseases, dyslipidemia, changes in corporal composition, or immunological disorders [209,210]. Thus, oxidative stress and inflammation observed during menopause and its stages might be effectively modulated by dietary supplementation with SOD-rich *T. chuii* via activation of the NRF2/SIRT1 pathway, which could promote health benefits through achieving a better redox balance. However, further research and clinical trials are required to evaluate the potential benefits of SOD-rich *T. chuii* for women’s health applications.

Although SOD-rich *T. chuii* supplementation may hold particular promise for individuals with a more pro-oxidative or pro-inflammatory state, potential benefits in already healthy individuals wishing to use the ingredient as part of a healthy and active lifestyle should not be discounted. Indeed, SOD-rich *T. chuii* has already been reported to elicit beneficial effects in such individuals (Table 2). A compromised redox state, which can result from poor lifestyle choices (e.g., smoking, imbalanced diet, and/or sedentary behavior) can lead to cell damage as a consequence of oxidative stress and inflammation [211,212,213]. Therefore, dietary interventions with SOD-rich *T. chuii* might help to prevent health challenges in many individuals who do not meet physical activity and dietary intake guidelines.

## Figures and Tables

**Table 1:** Published studies reporting the effects of SOD-rich *T. chuii* in rodent models.

Strain	N	Age	Dose (mg/Kg/day)	Duration	Main Outcomes	Reference
Wistar (male)	28	8 weeks old	2.55/5.1	6 weeks	↑ SOD, GPx and CAT intramuscular activity ↓ Serum MDA and myoglobin ↓ Serum CK activity	[16]
Wistar (male)	28	8 weeks old	2.55/5.1	6 weeks	↑ Muscle MyoD, NCAM ↓ Muscle myostatin, MAFbx, MuRF-1 ↓ Serum CK activity	[20]
Wistar (male)	28	8 weeks old	2.55/5.1	6 weeks	↓ Muscle IL-1β and TNFα ↓ Serum IL-1β and TNFα ↓ Muscle MDA	[21]
Sprague-Dawley (male)	50	7 weeks old	0.17/1.7/17	8 weeks	↓ Serum oxLDL ↑ Serum IL-10 ↑ Liver GPx activity ↑ Liver GSH content Gene expression in liver: ↑ GPx1, GSR, GSH-S, SOD1, SOD2, GCLM ↓ TGF-β1, NF-κB1 Gene expression in MWAT: ↑ ACDC, IL-10 ↓ IL-1β, TNFα, IFNγ Gene expression in thymus: ↑ IL-10 ↓ IL-1β, TNFα, IFNγ, NF-κB1 Gene expression in spleen: ↑ IL-10 ↓ IL-1β, IFNγ, NF-κB1	[15]

ACDC: adiponectin; CAT: catalase; CK: creatine kinase; GCLM: glutamate-cysteine ligase modifier subunit; GPx: glutathione peroxidase; GPx1: glutathione peroxidase 1; GR: glutathione-disulfide reductase; GSH: glutathione; GSH-S: glutathione synthetase; IFNγ: interferon γ; IL-1β: interleukin-1β; IL-10: interleukin-10; MAFbx: muscle atrophy F-box; MDA: malondialdehyde; MuRF-1: muscle RING-finger protein-1; MWAT: mesenteric white adipose tissue; MyoD: myogenic differentiation factor; N: number of animals; NCAM: neural cell adhesion molecules; NF-κB1: nuclear factor kappa B subunit 1; oxLDL: oxidized low-density lipoprotein; SOD: superoxide dismutase; SOD1: superoxide dismutase 1; SOD2: superoxide dismutase 2; TGF-β1: transforming growth factor-β1; TNFα: tumor necrosis factor-α; *T. chuii*: *Tetraselmis chuii*. ↑ represents an increase in parameters or in expression of target genes, whereas ↓ represents a decrease in parameters or down-regulation of target genes.
